# Interview with Ruotong Chen, recipient of the 2026 Epilepsia Open Prize for Clinical Research

**DOI:** 10.1002/epi4.70321

**Published:** 2026-08-13

**Authors:** Merab Kokaia, Piero Perucca

**Affiliations:** ^1^ Faculty of Medicine, Epilepsy Center Lund University Lund Sweden; ^2^ Department of Medicine The University of Melbourne Melbourne Victoria Australia; ^3^ Bladin‐Berkovic Comprehensive Epilepsy Program Austin Health Melbourne Victoria Australia

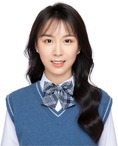

Ruotong Chen discusses the award‐winning research, its impact, and future directions.

## 1 PLEASE TELL US ABOUT YOURSELF, INCLUDING YOUR EDUCATION, TRAINING, AND CURRENT POSITION

I'm currently working on my PhD in Neurology at the Second Affiliated Hospital of Zhejiang University School of Medicine, under Professor Shuang Wang. Before that, I had 3 years of clinical practice. My clinical training has focused on epilepsy, mostly diagnosis and management. Along the way, I've developed a particular interest in neuroimaging post‐processing and neuropsychological assessment. It's been a rewarding mix of brain science and hands‐on patient work.

## 2 HOW DID YOU BECOME INTERESTED IN CONDUCTING RESEARCH IN THIS FIELD?

Our research group has long been dedicated to the study of temporal lobe epilepsy (TLE). Temporal lobe epilepsy with hippocampal sclerosis (TLE‐HS) is a major subtype of TLE, characterized by a high rate of drug resistance and often accompanied by memory decline. In recent years, patients with no lesions on magnetic resonance imaging (TLE‐NL) have also gained increasing attention. These two subtypes differ significantly in clinical presentation, age of onset, febrile convulsion history, and cognitive profiles. However, the distinct pathogenesis of TLE‐HS and TLE‐NL is poorly understood.

The glymphatic system (GS) is a recently identified waste clearance pathway in the brain and is thought to play a critical role in cognitive function. Growing evidence has demonstrated glymphatic dysfunction in several epilepsy types. However, at the time, we designed our study, the role of the glymphatic system in patients with TLE‐HS or TLE‐NL was unexplored. In particular, whether glymphatic dysfunction contributes to the distinct clinical and cognitive profiles of TLE‐HS and TLE‐NL, and whether it correlates with memory impairment in these patients, were unanswered questions. This gap in knowledge motivated me to pursue this research direction.

## 3 DESCRIBE THE QUESTION YOUR STUDY ADDRESSED AND HOW YOU APPROACHED IT

Our study aimed to answer three key questions: (1) Do the two major subtypes of TLE (TLE‐HS and TLE‐NL) exhibit glymphatic dysfunction, and if so, do the patterns of impairment differ between them? (2) Are there associations between glymphatic function and other clinical characteristics (such as seizure frequency, history of focal to bilateral tonic–clonic seizures, or drug resistance) in TLE patients? (3) Is there a correlation between glymphatic function and memory performance in TLE patients?

Methodologically, we employed the diffusion tensor image analysis along the perivascular space (DTI‐ALPS) index, a validated non‐invasive neuroimaging marker, to reflect glymphatic function. We compared the DTI‐ALPS index among three groups: healthy controls, TLE‐HS patients, and TLE‐NL patients. Memory performance was assessed using the Wechsler Memory Scale‐Revised (Chinese version). We then analyzed the relationships between the DTI‐ALPS index and various clinical factors, as well as memory scores.

## 4 WHAT WERE THE KEY FINDINGS, AND HOW DO YOU INTERPRET THEM?

Our study yielded several important findings. First, glymphatic impairment was observed in both TLE‐HS and TLE‐NL, but with distinct patterns: TLE‐HS patients showed bilateral DTI‐ALPS reduction (more severe ipsilaterally), whereas TLE‐NL patients exhibited impairment restricted to the ipsilateral hemisphere. This differential pattern may partially explain the clinical differences between the two subtypes, including the more severe and widespread cognitive deficits seen in TLE‐HS. Second, in the TLE‐NL group, a history of focal to bilateral tonic–clonic seizures was associated with more severe and bilateral glymphatic dysfunction, suggesting that high‐intensity seizures may contribute to long‐term glymphatic impairment. Third, and most notably, the reduced DTI‐ALPS index was significantly correlated with memory impairment in TLE‐HS patients, independent of hippocampal volume. Specifically, multiple regression analysis showed that the average DTI‐ALPS index accounted for approximately 11% of the variance in memory quotient beyond that explained by ipsilateral hippocampal atrophy. These findings suggest that targeting glymphatic clearance may represent a novel therapeutic strategy for memory improvement in epilepsy.

## 5 WHAT ARE THE NEXT STEPS FOR YOUR RESEARCH, AND WHAT ARE YOUR BROADER CAREER GOALS?

In our future work, we plan to:

- Establish a longitudinal cohort, enabling us to track dynamic changes in glymphatic function, seizure burden, and cognitive performance over time. Compared with assessing whether a patient's memory has declined relative to their peers, the rate of decline relative to their own baseline is far more meaningful.
- Incorporate multimodal glymphatic assessment, as DTI‐ALPS alone does not fully capture global glymphatic dynamics. We plan to integrate additional imaging metrics, including choroid plexus volume, DTI‐based estimated perivascular space diffusion (DTI‐EstPVeD), and global BOLD‐CSF coupling. These approaches will provide a more comprehensive understanding of neurofluid dysregulation in TLE.

## 6 WHAT DOES RECEIVING THE EPILEPSIA OPEN PRIZE FOR THE BEST CLINICAL RESEARCH ARTICLE PUBLISHED IN 2025 MEAN TO YOU, YOUR TEAM, AND YOUR FUTURE WORK?

Receiving the Epilepsia Open Prize is a tremendous honor and a profound encouragement, both for me personally and for our entire laboratory. It serves as a strong validation of the phase‐specific work we have accomplished over the past several years. For our institution, this award highlights the high‐quality epilepsy research being conducted at the Second Affiliated Hospital of Zhejiang University and strengthens our collaborations across departments, including neurology and radiology. For my future work, this recognition motivates me to continue our in‐depth investigation of temporal lobe epilepsy, always guided by patient needs.

This prize is not an endpoint, but rather a milestone that energizes us to push further. Thank you to Epilepsia Open and the ILAE for this recognition. We look forward to doing more for the epilepsy community.

## CONFLICT OF INTEREST STATEMENT

None of the authors has any conflict of interest to disclose.

## Data Availability

Data sharing not applicable to this article as no datasets were generated or analysed during the current study.

